# Supplementary material for: Exploring Localized Provoked Vulvodynia: Insights from Animal Model Research
Source: Int J Mol Sci. 2024 Apr 11;25(8):4261. doi: 10.3390/ijms25084261 (PMC11050705; doi:10.3390/ijms25084261)
Supplement: Supplementary file 1 [file ijms-25-04261-s001.zip › ijms-2952237-supplementary.pdf]

Supplementary material:

S1. Involvement of fibroblasts, PGE, IL6:

Fibroblasts play an important role in the immune response[113]. SPMs are chemicals that modulate and fasten inflammation resolution in many chronic pain disorders. [114,115] Previous animal studies have shown that fibroblasts from women with LPV, show high level of production of IL-6 and PGE2. Furthermore, In SPMs mice models efficacy of marine 1 in modulating inflammation cascade, reduction in IL-6 and PGE2 and increasing pain thresholds and enhancing neuropathic pain have been depicted. [116–118] All SPMs have an analgesic effect [119].

In few of the fibroblasts studies a study of three-dimensional (3D) tissue was used. 3D enables investigation of the physiological function of a tissue.[120] Culture model of 3D fibroblasts from mice vulvar tissue showed that the polysaturated fatty acids, DHA and marine 1 are SPMs that were effective in reducing PGE2 and IL-6 levels in fibroblasts. [14]

S2. Involvement of local renin-angiotensin system (RAS):

T and B cells and macrophages can display RAS elements. [69] Angiotensinogen (AGT) is a precursor protein of all angiotensin peptides. AGT is cleaved by renin to provide angiotensin I (AGTI). The conversion of AGTI to AGTII is mediated by peptidases such as angiotensin-converting enzyme (ACE), cathepsin G and chymase. [69,121]

Prior animal model studies have shown that inflammatory hypersensitivity and hyperinnervation occur in concert with the activation of the local renin-angiotensin system (RAS).[69] studies shows that AT2 increases nerve growth. Blocking the effects of angiotensin II (AGTII)on AT2 receptors such with PD, decreases the mechanical hypersensitivity and sensory axon sprouting. [122–124]

The accumulation of immune cells, T and B cells and macrophages in vulvar tissue from women diagnosed with vulvodynia have also been reported [125].
